# Role of Primary Health Care in child hospitalization due to pneumonia: a case-control study**^1^**

**DOI:** 10.1590/1518-8345.1731.2892

**Published:** 2017-05-22

**Authors:** Juliana Coelho Pina, Suzana Alves de Moraes, Isabel Cristina Martins de Freitas, Débora Falleiros de Mello

**Affiliations:** 2 PhD, Adjunct Professor, Departamento de Enfermagem, Centro de Ciências da Saúde, Universidade Federal de Santa Catarina, Florianópolis, SC, Brazil.; 3 PhD, Associate Professor, Escola de Enfermagem de Ribeirão Preto, Universidade de São Paulo, PAHO/WHO Collaborating Centre for Nursing Research Development, Ribeirão Preto, SP, Brazil.; 4 PhD, Social scientist.

**Keywords:** Child, Pneumonia, Hospitalization, Primary Health Care, Risk Factors, Protective Factors

## Abstract

**Objective:**

to evaluate the association of primary health care and other potential factors in relation to hospitalization due to pneumonia, among children aged under five years.

**Method:**

epidemiological study with a case-control, hospital-based design, which included 345 cases and 345 controls, matched according to gender, age and hospital. Data were collected using a pre-coded questionnaire and the Primary Care Assessment Tool, analyzed by means of multivariate logistic regression, following the assumptions of a hierarchical approach.

**Results:**

the protective factors were: family income >US$216.12 (OR=0.68), weight gain during pregnancy ≥10 kg (OR=0.68), quality of Primary Health Care (OR for scores >3.41=0.57; OR for scores >3.17 and ≤3.41=0.50), gastro-esophageal reflux (OR=0.55), overweight (OR=0.37) and birth interval ≥48 months (OR=0.28). The risk factors included: parity (2 childbirths: OR=4.60; ≥3 childbirths: OR=3.25), out-of-date vaccination (OR=2.81), undernutrition (OR=2.53), history of wheezing (≥3 episodes OR=2.37; 1 episode: OR=2.13), attendance at daycare center (OR=1.67), and use of medicines over the past month (OR=1.67).

**Conclusion:**

primary health care and its child health care practices, such as nutritional monitoring, immunization, care to prevalent illnesses, prenatal care and family planning need to be prioritized to avoid child hospitalization due to pneumonia.

## Introduction

The importance of pneumonia to child morbidity and mortality has been widely addressed in the literature^1^ ^-^ ^2^. The most recent world estimate for children aged under five years is 120 million new episodes per year, along with severe cases and high rates of hospitalizations^2^. In Brazil, 1.5 million new cases of community-acquired pneumonia (CAP) occur in this age group every year, and the disease ranks first on the list of hospitalization causes of these children in all regions of the country^3^.

Knowledge of the risk factors for hospitalization due to pneumonia makes it possible to identify priorities for preventing and managing this disease. These factors are: gender, age, type of birth, birth weight, gestational age, birth order, breastfeeding, malnutrition, previous and current morbidities, immunization, use of medications, parent and socioeconomic characteristics, basic sanitation, indoor and outdoor air pollution, child care outside the home and access to health care services^1^.

Primary Health Care (PHC) can be considered as a factor that influences hospitalization due to pneumonia. The role of PHC has been investigated in relation to controlling pneumonia in regard to having access to health care services and clinical management of the disease^4^. PHC is the level of health care that is focused on early diagnosis, treatment and interventions, the purpose being to decrease exposure to risk factors and/or increase exposure to protective factors associated with pneumonia^3^. The inclusion of PHC as an explanatory factor of this disease should take all of the characteristics of its health actions into consideration, guided by the following attributes: first contact access, longitudinality, comprehensiveness, health care coordination, family orientation and community orientation^5^. It is important to take not only the care given during the acute episodes into account, because factors related to health care and health communication provided routinely for child, mother and family can be reflected in child health^6^.

Factors involved in child hospitalization due to pneumonia can represent interventions by professionals working in PHC (such as immunization, nutritional counseling, preventive care at home); in addition, aspects related to family income and parents' education can influence access to PHC services which, in turn, can influence the risk of hospitalization due to the disease^1^. This understanding supports the construction of a hierarchical conceptual framework for child hospitalization due to pneumonia, which is based on methods with a hierarchical approach to evaluate the relationships between exposure and outcome, considering that the risk factors can be influenced by those located in preceding levels, either directly or indirectly^7^. From a hierarchical point of view, however, we did not find any studies that included PHC as an explanatory variable for hospitalization due to pneumonia.

Thus, this study aimed to evaluate the association of PHC and other potential factors in relation to hospitalization due to pneumonia, among children aged under five years .

## Method

This is an analytical epidemiological study with a hospital-based case-control design. The study was developed at three public hospitals in the city of Ribeirão Preto, Brazil.

Brazil is a South American developing country with a unified free-of-charge public health care system. The Southeast is home to 43% of Brazil's population and accounts for 56% of the Gross Domestic Product (GDP). Ribeirão Preto has the tenth largest GDP in the state of São Paulo. The weather is characterized by one mild, cool and dry and another hot and wet season, with moderately high temperatures^8^.

The study was developed at three hospitals within the framework of the Brazilian Unified Health System - SUS (Hospital A - state institution, national reference for clinical specialties, pediatric clinic with 58 beds; Hospital B - municipal institution, 18 pediatric beds; Hospital C - municipal institution, 20 pediatric beds). The study was conducted only with children living in Ribeirão Preto, users of the primary health care network through the SUS.

### Selection of Cases and Controls

With the aim of detecting OR ≥2.0 (two-tailed tests), with a statistical power of 80% and α=0.05, and considering 10% as the lowest probability of exposure among the controls, a total of 345 cases and 345 controls were selected. Cases were children aged under five years who had been hospitalized due to CAP, as confirmed through radiological examination, at the participating hospitals. Each case was arranged according to gender and age group (2-6; 6-12; 12-24 and 24-60 months) with a control in the same hospital. Minimizing Berkson's bias involved including incident cases and selecting hospital controls with a variety of admission diagnoses, including those with diseases of the upper respiratory tract.

Living in the city for less than six months, having a recent history of liquid or foreign body aspiration (due to the diagnostic hypothesis of aspiration pneumonia), and being aged under two months were used as exclusion criteria for cases and controls. Among the controls, children were also excluded if there was a suspected or current diagnosis of pneumonia, if they had some degree of kinship or if they lived in the same home with these cases.

The data were continuously collected between March 2012 and August 2013, taking into account the seasons of the year. The interviewers visited the hospitals daily, recruiting patients according to either order of hospitalization or order of arrival. In situations where there was patient refusal or exclusion, another eligible child was identified, while adhering to the same order. Interviewers trained with regard to anthropometrics and blinded to the research objectives collected the data. A precoded questionnaire was used, containing all the variables of the study, except the variable *quality of PHC*, collected by the Primary Care Assessment Tool (PCA Tool) - child version^9^. This instrument measures the quality of PHC from the user´s perspective, based on the presence and extent of four essential attributes (first contact access, longitudinality, comprehensiveness and coordination of health care) and of two derived attributes (family and community orientations). The tool has a 4-point Likert-type scale for each area, which makes it possible to calculate the scores relating to each attribute (mean of the responses to the items of the area). Scores >3 indicated a strong presence and extent of the attribute or set of attributes evaluated^9^. The essential PHC score (mean of the essential attribute items) and the overall PHC score (mean of the essential and derived attribute items) were obtained^9^. The parents or legal guardians of the child answered all questions from the PCA Tool and those included in the precoded questionnaire. Therefore, all the explanatory variables of the study were collected during the interviews, except the anthropometric measurements, which were collected as described below.

For children aged under two years, weight was measured using an infant digital scale (precise to 5 grams) and height was measured using a horizontal stadiometer. For children aged older than two years, a portable digital scale (100-gram precision) and a vertical stadiometer were used. The techniques for anthropometric measurement and the cut-offs of the anthropometric indexes followed World Health Organization (WHO) recommendations.

**Figure 1:**
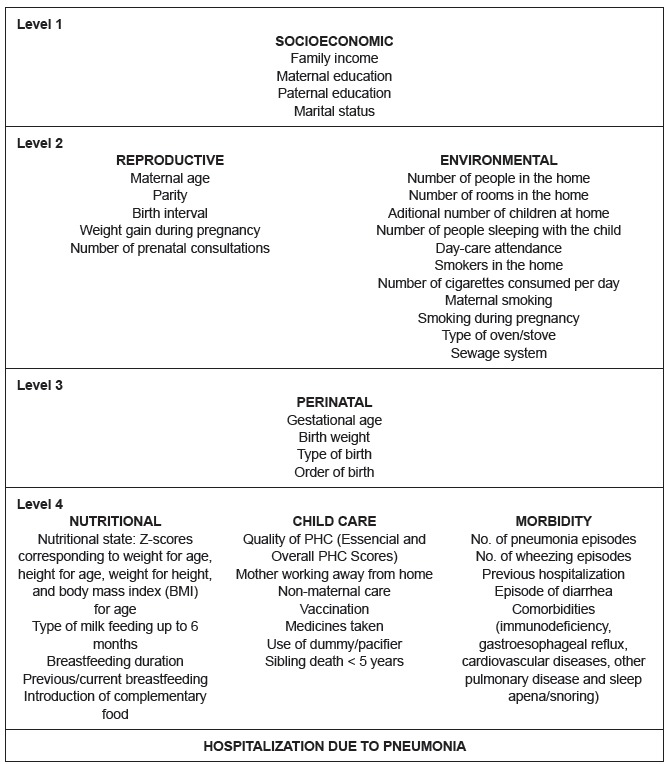
Conceptual hierarchical model of the potential factors associated with hospitalization due to pneumonia, among children aged under five years

A conceptual hierarchical model (Figure 1) was composed, based on the literature^7^, including the outcome and the explanatory variables, which guided the statistical techniques for data analysis.

Some study variables require further explanation regarding their definition and classification, such as: *Family income* (monthly income of the inhabitants of the same home, US dollars according to tercile distribution), *Weight gain during pregnancy* (mother's weight during the period before pregnancy and at the time of the birth, continuous variable <10 or ≥10kg), *Smokers in the home* (presence/absence of smokers at home), *Smoking during pregnancy* (binary variable), *maternal smoking* (non-smoker, former smoker, or smoker), *Gestational age* (<37 or ≥37 weeks), *Birth weight* (<2500 or ≥2500g), *Nutritional state* (Z-scores weight for age, height for age, weight for height, as ≥-2.0 or <-2.0, and Body Mass Index (BMI) for age: eutrophic, underweight, or overweight), *Type of milk feeding up to six months* (exclusive breastfeeding, predominantly breastfeeding, mixed milks, and formula feeding), *Breastfeeding duration* (months according to tercile distribution), *Previous/current breastfeeding* (never breastfed, previously breastfed, currently breastfed), *Introduction of complementary food* (did not introduce, before 6 months, at 6 months, or after 6 months), *Quality of PHC* (Overall and Essential PHC Scores, according to quartile distribution), and *Non-maternal care* (grandmother, daycare, school, or other).

### Statistical Analysis

Cases and controls were characterized according to sociodemographic variables. The groups were compared using the chi-square likelihood ratio. During the analytical phase, crude and adjusted Odds Ratios (OR), with their respective 95% confidence intervals, were estimated using non-conditional multivariate logistic regression^10^, following the assumptions of hierarchical models. Through univariate models, the inclusion criterion for variables in the subsequent models was taken as *p* values <0.25 for the Wald test. During the next stage, the variables that remained in the model presented *p*<0.10. The final model was composed of variables with *p*<0.05. The order of entry for the variables in the multivariate models was defined according to the conceptual framework (Figure 1), beginning from the first hierarchical level and thus leading to simultaneous inclusion of variables at the same level, while always being adjusted for the variables of the preceding levels^10^. The use of unconditional analysis was based on two assumptions: 1) the results were very close to those obtained in the conditional analysis; and 2) matching according to gender and age group does not characterize close matching^10^. The goodness of fit of the final model was evaluated based on the Hosmer-Lemeshow chi-square test^10^, while the sensitivity (se) and specificity (sp) were obtained from the ROC curve. The results were: chi-square=12.30 with df=8 and p value=0.1382; Se=70.48%; Sp=74.30%; and accuracy=0.7835. All the analyses were run in Stata version 12.

 This study received approval from the Research Ethics Committee of the University of São Paulo at Ribeirão Preto College of Nursing (date: 11/29/2011; protocol number: 1404/2011), following the recommendations set out in the Declaration of Helsinki and Resolution 196/96 of the National Health Council.

## Results

The sociodemographic characteristics of the study population are presented in Table 1.

**Table 1:** Characteristics of cases and controls according to sociodemographic variables. Ribeirão Preto, SP, Brazil, 2013

Characteristics		Cases		Controls		ᵡ**^2^** *
		n	%	n	%	
Gender						0.939
	Male	179	51.88	179	51.88	
	Female	166	48.12	166	48.12	
Age range (months)						0.990
	02-05.9	77	22.32	76	22.03	
	06-11.9	82	23.77	85	24.64	
	12-23.9	98	28.41	95	27.54	
	24-59.9	88	25.51	89	25.80	
Family income in tertiles (US$)**^†^**						0.113
	1**^st^** tertile (≤123.50)	133	38.55	109	31.59	
	2**^nd^** tertile >123.50 and ≤216.12	108	31.30	111	32.17	
	3**^rd^** tertile >216.12	104	30.14	125	36.23	
Maternal education (years)						0.321
	0-4	31	8.99	30	8.70	
	5-8	133	38.55	115	33.33	
	≥ 9	181	52.46	200	57.97	
Paternal education (years)						0.705
	0-4	43	14.05	42	13.82	
	5-8	132	43.14	122	40.13	
	≥ 9	131	42.81	140	46.05	
Marital status						0.731
	With partner	254	73.62	250	72.46	
	No partner	91	26.38	95	27.54	
Mother working away from home						0.445
	No	190	55.1	180	52.2	
	Yes	155	44.9	165	47.8	
Maternal age (years)						0.771
	< 20	27	7.8	30	8.7	
	20-34.9	259	75.1	250	72.7	
	≥35	59	17.1	64	18.6	
No. of rooms in the home						0.693
	≤ 3	65	18.8	61	17.7	
	≥ 4	280	81.2	284	82.3	
No. of people in the home						0.048
	< 4	73	21.2	99	28.7	
	4-5	171	49.6	164	47.5	
	≥ 6	101	29.3	82	23.8	

* Likelihood ᵡ^2^, considering the respective degrees of freedom.

† US Dollar exchange rate on 06/29/2016: 3.29.

There was a greater proportion of controls with a higher family income and parent education level, although these differences were not statistically significant. There were no significant differences either between the two groups regarding maternal age, marital status or whether the mother worked away from home. There was a greater proportion of homes with six or more people living in the same home. Regarding the number of rooms in the home, however, there was no significant difference between the groups. Table 2 shows the results related to the final model.

**Table 2:** Crude and adjusted odds ratios and confidence intervals (95%) - Final model considering the hierarchical approach. Ribeirão Preto, SP, Brazil, 2013

Hierarchical levels			Crude OR* (CI95%)	Adjusted OR* (CI95%)
1^st^ - Socioeconomic Variables				
	Family income in tertiles (US$)^†^			
		1^st^ tertile (≤123.50)	1.00	1.00
		2^nd^ tertile >123.50 and ≤216.12	0.80 (0.55 to 1.15)	0.80 (0.55 to 1.15)
		3^rd^ tertile >216.12	0.68 (0.47 to 0.98)	0.68 (0.47 to 0.98)
				
2º - Reproductive and Environmental Variables**^‡^**				
	Parity (number of births)			
		1	1.00	1.00
		2	1.58 (1.09 to 2.28)	4.60 (2.18 to 9.72)
		≥ 3	1.38 (0.96 to 1.99)	3.25 (1.55 to 6.81)
	Birth interval (months)			
		< 24	1.00	1.00
		24-47	1.56 (1.02 to 2.39)	0.51 (0.25 to 1.05)
		≥ 48	0.86 (0.62 to 1.20)	0.28 (0.14 to 0.56)
	Weight gain during pregnancy (Kg)			
		< 10	1.00	1.00
		≥ 10	0.69 (0.48 to 0.97)	0.68 (0.47 to 0.97)
	Day-care attendance			
		No	1.00	1.00
		Yes	1.60 (1.17 to 2.19)	1.67 (1.16 to 2.41)
				
3**^rd^** - Nutritional, child care and morbidity variables**^§^**				
	Z-score of BMI**^‖^** for age			
		Eutrophic	1.00	1.00
		Underweight	1.99 (1.02 to 3.85)	2.53 (1.06 to 6.05)
		Overweight	0.42 (0.21 to 0.81)	0.37 (0.14 to 0.99)
	Essential PHC**^¶^** Score			
		1**^st^** quartile (≤2.79)	1.00	1.00
		2**^nd^** quartile (> 2.79 e ≤3.17)	0.79 (0.53 to 1.18)	0.98 (0.57 to 1.68)
		3**^rd^** quartile (>3.17 e ≤ 3.41)	0.50 (0.32 to 0.76)	0.50 (0.28 to 0.88)
		4**^th^** quartile (>3.41)	0.56 (0.37 to 0.86)	0.57 (0.32 to 0.99)
	Vaccination			
		Up-to-date	1.00	1.00
		Delayed	2.11 (1.49 to 2.98)	2.81 (1.76 to 4.49)
	Medicines taken			
		No	1.00	1.00
		Yes	1.60 (1.10 to 2.32)	1.67 (1.00 to 2.78)
	Number of wheezing episodes			
		0	1.00	1.00
		1	1.98 (1.33 to 2.94)	2.13 (1.31 to 3.47)
		2	1.36 (0.79 to 2.33)	1.04 (0.53 to 2.06)
		≥ 3	2.79 (1.78 to 4.36)	2.37 (1.35 to 4.15)
	Gastroesophageal reflux			
		No	1.00	1.00
		Yes	0.72 (0.46 to 1.13)	0.55 (0.31 to 0.99)

* Odds ratio. † US Dollar exchange rate in 06/29/2016: 3.29. ‡ Adjusted for family income. § Adjusted for level 1 and 2 variables. ‖ Body Mass Index. ¶ Primary Health Care.

The quality of PHC demonstrated an association with child hospitalization due to pneumonia through the *essential PHC score*, which measures the presence and extent of the health care attributes (first contact access, longitudinality, comprehensiveness and coordination). In addition, factors related to family income, maternal obstetric history, child and mother nutritional status, morbidity and child health care were associated with hospitalization due to pneumonia in the population studied.

## Discussion

This study serves to fill a gap in the literature, demonstrating the contribution of PHC to prevent child hospitalization due to pneumonia. Therefore, the study goes beyond the access received during the disease, an aspect other authors have already pointed out^4^. The association identified in relation to the essential PHC score signals the importance of PHC organization as a regular source of health care^5^ ^,^ ^9^, and the link over time through a variety of articulated services and actions for children and their families, which increase the continuity of health care.

In relation to the ten variables that composed the final hierarchical model, eight are related to PHC service actions, especially care in child health, prenatal care and family planning. Furthermore, the principle of longitudinality and the complementarity of PHC actions between the child and women's health programs are relevant, as they monitor the health of children, women and families^11^. In childcare, the systematic follow-up of vaccination, child nutrition, childhood illnesses and habits, such as the use of medication is carried out ^1^ ^,^ ^11^, which contributes to improving integral child care.

One of the most important strategies for controlling pneumonia within PHC is vaccination^1^ ^,^ ^12^. In this study, situations in which out-of-date vaccination was used were seen more frequently among the cases, and these lead to a higher risk of hospitalization due to pneumonia. Vaccines against the influenza virus (*Haemophilus influenza type b*) and against *Streptococcus pneumoniae* grant specific protection^1^. Also, adequate immunization is considered as a proxy for childcare. Strategies need to be implemented to vaccinate all children who visit health services for other interventions ^13^.

In relation to nutritional variables, there was a higher risk of hospitalization due to pneumonia among children who were undernourished and a lower risk among overweight children. The effect of undernourishment on the occurrence and severity of pneumonia has already been reported in the literature^14^. Pneumonia, undernutrition and micronutrient deficiencies have common risk factors, such as not exclusively breastfeeding infants (< 6 months), zinc deficiency, and measles infection^1^ ^-^ ^2^.

A protective effect of being overweight against pneumonia among adults has previously been described^15^. Although there is evidence that obese individuals are more susceptible to inflammatory and viral pulmonary diseases, the impact of obesity on bacterial pneumonia is still uncertain^16^. These findings suggest that it might be a mechanism to explain the protective effect of obesity against those pneumonias. In this study, despite the aforementioned evidence from the literature, it is important to highlight that the abrupt acute weight loss among the cases, caused by lack of appetite and dehydration secondary to the respiratory condition, may have contributed towards the greater proportion of controls with overweight individuals and a consequent inverse association between obesity and child hospitalization due to pneumonia.

Regarding prevalent childhood illnesses, in this study, a history of wheezing was associated with the outcome studied, thus demonstrating the importance of identifying and following up individuals with recurrent wheezing, who might be undiagnosed as asthmatic individuals. Asthma leads to greater vulnerability to the pathogens that cause pneumonia, which in turn exacerbates asthmatic symptoms and contributes towards a worsening of the clinical conditions and the need for hospitalization^17^.

Also in relation to prevalent illnesses, there was a negative association observed between gastroesophageal reflux and child hospitalization due to pneumonia. There is evidence that chronic aspiration or microaspiration of gastric content may cause pulmonary harm, thus contributing to the genesis of aspiration pneumonia^18^. Reflux is not necessarily associated with aspiration or complications though, due to the protective mechanism of the laryngeal chemoreflex, which is triggered by the presence of acidic solutions (such as gastric juice) in the larynx^19^. Since the acidity of the gastric juice forms part of the individuals' innate immunity, small amounts of the liquid in the pharynx, without any associated aspiration, could neutralize some pathogens that cause CAP, thus contributing to the protective effect found. In this study, children with a recent history of liquid or foreign body aspiration were excluded. Thus, excluding children with a hypothesis of aspiration pneumonia may have led to a better characterization of the role of reflux in non-aspiration pneumonia. Despite the biological plausibility of this finding, one important limitation was that the maternal report of the health condition may have contributed towards potential errors in classifying the disorder. Future investigations with greater diagnostic accuracy for gastro-esophageal reflux could elucidate its effect on non-aspiration pneumonia.

Another factor relating to childcare that increased the risk of hospitalization due to pneumonia was the use of medicines during the 30 days prior to hospitalization. PHC professionals should analyze any abusive use of medicines in pediatrics. Non-recommended antibiotics predispose to bacterial resistance, while antipyretic drugs, acid-suppressants and glucocorticoids may interfere with the immune system, thereby increasing susceptibility to pneumonia^20^. However, one limitation in this study was that the medicines used were not specified. Thus, new studies designed to provide this information would be extremely useful to verify this discovered association.

Child nutritional status is influenced by current and previous nutrient intake, which includes the prenatal period. Multiparity and short intervals between pregnancies cause damage to the maternal body and have an influence on weight gain during pregnancy, which may result in low weight at birth and during childhood^21^. In this study, the variables *parity, birth interval, weight gain during pregnancy* and *birth weight* were associated with the outcome in the univariate analysis. In the multivariate model, however, low birth weight lost its statistical significance. Thus, the results suggest that the maternal reproductive factors acts, at least in one way, independently from the perinatal factors (such as the birth weight), possibly because the ability for maternal self-care would positively influence childcare^21^.

During the PHC activities, in addition to those in childcare, prenatal follow-up permits adequate weight gain during pregnancy, and care for women of reproductive age promotes family planning actions^11^, thereby providing guidance regarding parity and the birth interval, which are variables associated with the outcome studied.

The results of this study indicate attendance at a daycare center as a risk factor for the outcome, and this finding was consistent with the literature^22^. The concentration of children within these institutions may facilitate disease transmission and result in permanent exposure to pathogens that may cause repetitive episodes, thereby contributing towards worsening the medical condition and exacerbating the need for hospitalization^22^. Attendance at a daycare center is not directly the object of attention for the PHC professionals, but it is extremely important to articulate these two scenarios (health and childhood education) for child care, health promotion and disease prevention.

Another variable that composes the final model of this study, and which tends to go beyond the scope of the health sector, is family income. The effect of family income on the outcome studied was independent of marital status and parents' education, which is consonant with another study^23^. It should be pointed out that the family needs and income deserve to be seen in an intersectoral way. Intersectoral collaboration and resource mobilization are among the priority actions needed to achieve the goal of ending preventable deaths from pneumonia^24^.

Social inequalities lead to differences in exposure to risk factors and in access to effective interventions in pneumonia^2^ ^,^ ^12^. These differences may exist within the same community, which seems to be the case in our study population. Although the study region is among the most prosperous in the country^8^, low income was commonly identified among the participants, but especially among the cases, thereby contributing towards greater risk of hospitalization due to pneumonia than other diseases. These results corroborate the hypothesis that pneumonia is the disease that most reflects the effects of social inequalities. The expansion of the post-pneumococcal conjugate vaccine program and social improvements has improved child health in Brazil; however, these improvements were insufficient to overcome inequalities^12^.

Thus, the results of the this research highlight the contribution of the actions developed in PHC to the prevention of child hospitalization due to pneumonia, which ratifies the logic of including this pathology in the Brazilian list of primary care sensitive conditions.

The literature supports the main findings of his study, but some limitations are due. Although PHC assessments made by means of the PCA Tool provide a broader perspective of PHC characteristics, it is important to emphasize that this instrument only considers the user's perspective. Moreover, there was a diversity of respondents - even though the interviews were primarily performed with the mother, she was not always available. Therefore, these findings need to be confirmed by means of the PCA Tool version applied to healthcare professionals. It should be mentioned that it is possible that not all of children had access to the same PHC procedures, as there are different units within the same city (Family Health Unit, Basic Health Unit and/or Community Health Workers Program). This diversity can lead to different experiences with PHC for evaluation from the user's perspective. This analysis goes beyond the scope of this study though.

In addition, information on some previous exposures relating to the outcome, obtained from interviewees' reports, may have contributed towards a reverse causality and/or recall bias, among other problems. However, efforts were made to minimize these, such as the data collection inside the hospital instead of at home, ensuring the recruitment of incident cases and contemporary controls.

On the other hand, it should be emphasized that including children with other respiratory diseases among the controls would lead to the null hypothesis, due to the similarities between the two groups. Therefore, the strength of the associations found confirms the specificity of the effect of the exposures on hospitalization due to pneumonia, and not on other respiratory diseases.

The methodological rigor applied to the different stages of the study grants internal and external validity to the results, which can be generalized to similar populations in developing countries with the same PHC configuration. Replication studies in different contexts could contribute to further generalizing these results, especially for populations with distinct health systems. Comparative studies, conducted across developed and developing countries, could lead to a better understanding of the interaction between sociocultural characteristics and other factors involved in hospitalization due to pneumonia in children aged under five years.

## Conclusion

PHC and its child health care practices, such as nutritional monitoring, immunization, attention to prevalent diseases, prenatal care and family planning, constitute actions to be prioritized so that child hospitalization due to pneumonia can be avoided.

The hierarchical approach permitted understanding the phenomenon of child hospitalization due to pneumonia in the Brazilian population studied. It depicted the influence of the characteristics related to child, mother and family, along with the care practices and qualified PHC. These findings should direct rational planning of actions to avoid child hospitalization, especially in the context of PHC.
